# Effect of functional fatigue on vertical ground reaction force among individuals with flat feet

**DOI:** 10.1186/1757-1146-5-S1-P5

**Published:** 2012-04-10

**Authors:** Sahar Boozari, Ali Ashraf Jamshidi, Mohammad Ali Sanjari, Hassan Jafari

**Affiliations:** 1 Department of Physical Therapy, Tehran University of Medical Sciences, Tehran, 1545913187, Iran; 2 Biomechanics Laboratory, Rehabilitation Research Center, Tehran University of Medical Sciences, Tehran, 1545913187, Iran

## Background

Flat foot as one of the lower extremity deformities might change some kinetic variables of gait. Fatigue can deteriorate the muscle ability in supporting joints and can alter the vertical ground reaction force (GRF) [1,2]. This study examined the fatigue effect on vertical GRF in individuals with flat feet compared with a normal group during barefoot walking.

## Materials and methods

Seventeen subjects with flat feet and 17 normal subjects completed the test. Three vertical GRF measures (F_1_; the first peak force, F_2_; minimum force; and F_3_; the second peak force) were extracted before and after a functional fatigue protocol. To check the homogeneity of the velocity among conditions, the average velocity of the anteroposterior center of pressure (COP_y_) excursion was calculated. A repeated measure ANOVA was conducted to analyze data.

## Results

**Figure 1:**
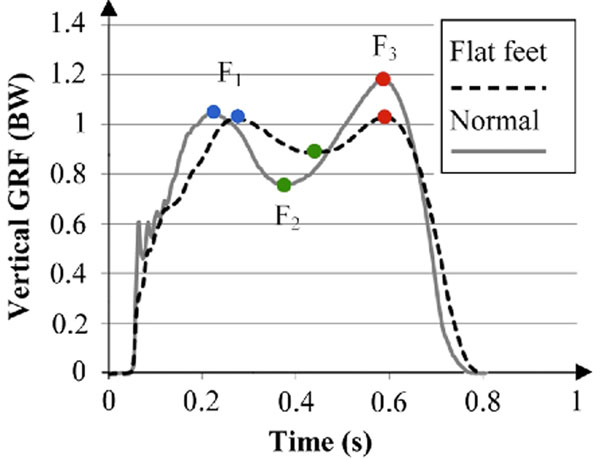
Vertical GRF curves for a subject with flat feet and a subject with normal feet after fatigue.

For the average COP_y_ velocity, no significant fatigue, group and interaction effects were seen. F_2_ was higher in the flat feet group compared with the normal group (*p* < 0.05). The fatigue protocol resulted in higher F_2_ and lower F_3_ in both groups (*p* < 0.05). See Figure 1. as the sample vertical GRF curves for a subject with flat feet and a subject with normal feet after fatigue.

## Conclusions

The higher F_2_ in the flat feet group, which results in a decrease drop in vertical GRF, might be due to more flexible foot joints. Foot muscles lose their appropriate ability to control the foot joints and MLA due to fatigue [2-4] which results in higher F_2_ for both groups. Furthermore the muscles could not make a proper lever arm for the propulsion gait phase after fatigue [2] resulting in lower F_3_ for both groups.
